# Effectiveness of Water Desalination by Membrane Distillation Process

**DOI:** 10.3390/membranes2030415

**Published:** 2012-07-17

**Authors:** Marek Gryta

**Affiliations:** West Pomeranian University of Technology, Szczecin ul. Pułaskiego 10, Szczecin 70-322, Poland; Email: marek.gryta@zut.edu.pl; Tel.: +48-91-449-47-30; Fax: +48-91-449-46-86

**Keywords:** membrane distillation, desalination, thermal efficiency

## Abstract

The membrane distillation process constitutes one of the possibilities for a new method for water desalination. Four kinds of polypropylene membranes with different diameters of capillaries and pores, as well as wall thicknesses were used in studied. The morphology of the membrane used and the operating parameters significantly influenced process efficiency. It was found that the membranes with lower wall thickness and a larger pore size resulted in the higher yields. Increasing both feed flow rate and temperature increases the permeate flux and simultaneously the process efficiency. However, the use of higher flow rates also enhanced heat losses by conduction, which decreases the thermal efficiency. This efficiency also decreases when the salt concentration in the feed was enhanced. The influence of fouling on the process efficiency was considered.

## 1. Introduction

Fresh water derived from sea water is produced by evaporation processes (e.g., multi-stage flash) or by membrane processes, such as reverse osmosis or electrodialysis. Water desalination can be also accomplished by membrane distillation (MD), which is an evaporation/condensation process of volatile components through a hydrophobic porous membrane [1,2]. The MD membranes are not selective and their pores are filled only by the gas phase. This creates a vapor gap between the feed and the produced distillate, which is usually in direct contact with the membrane [1,2,3,4]. This MD configuration is called Direct Contact Membrane Distillation (DCMD). 

The retention of the gas phase inside the membrane pores during the MD process is an essential condition for the process to proceed. The hydrophobic nature of the membrane prevents liquid solutions from penetrating into the pores due to surface tension forces. Membranes having these properties are prepared from polymers with a low surface energy, such as polypropylene (PP), polytetrafluoroethylene (PTFE) or poly(vinylidene fluoride) (PVDF) [1]. 

Microporous polypropylene membranes are commonly prepared by a thermally induced phase separation (TIPS) process. In this process, strongly related to temperature-dependent polymer solubility, the polymer is dissolved in an appropriate solvent at elevated temperature. When the temperature is decreased, at a certain temperature the polymer is no longer soluble, and phase separation is induced. After removing the solvent, the resulting pores giving the membrane its unique features [1]. Variation of membrane morphology and pore structure is possible by controlling the thermodynamic conditions of the process (concentration of the polymer) and the kinetics (temperature, residence and drawing-relaxation time). 

Microporous PVDF hollow fiber membranes are prepared by the dry/wet spinning or the wet spinning technique. This technique permits the preparation of asymmetric membranes. The different pore size and porosities are achieved by varying the dope composition and spinning conditions. Finger-like microvoids and a sponge-like pore network allow the holding of a large volume of air, which can reduce the heat lost by conduction through the membrane wall. It was found that the polymer dope composition was the most significant parameter controlling the morphology and permeation characteristic of PVDF membranes [1,5].

During the MD a part of the membrane pores may be made wet, and as a result, the wet membrane pore allows the passing of liquid to the other side of the membrane [6]. Therefore, the properties of membrane material and membrane porous structure are of crucial importance for MD process performance [1,2,3,4,5,6].

The MD separation mechanism is based on the vapor/liquid equilibrium of a liquid mixture, therefore, the permeate composition is dependent on the partial pressure of respective components of the feed. For solutions containing non-volatile solutes only the water vapor is transferred through the membrane; hence, the distillate obtained comprises demineralized water [1,2,3,4]. However, when the feed contains various volatile components, they are also transferred through the membranes to the distillate [1,7]. Based on this separation mechanism, the major application areas of MD include water desalination, the concentration of aqueous solutions and the separation of fermenting broth [1,2,3,4,5,6,7,8].

Similar to other distillation processes, the MD also requires energy for water evaporation. The total heat flux transferred through the membrane, typically 50%-80% is consumed as latent heat for permeate production, while the remainder is lost by thermal conduction. The heat loss decreases when the MD system works under high operating temperatures [1,2]. Unfortunately, the fouling intensity also increases with feed temperature [9]. 

The hydrodynamic conditions occurring in the membrane modules influence the heat and mass transfers, and have a significant effect on MD process efficiency. The most advantageous operating conditions of the MD module were obtained with the membranes arranged in the form of braided capillaries. This membrane arrangement improves the hydrodynamic conditions (the shape of braided membranes acts as a static mixer), and as a consequence, the module yield was enhanced [2,9,10]. The efficiency of MD process depends, to a significant degree, on the morphology of used membranes. The permeate flux increases along with an increase in the membrane porosity, pore diameter and is inversely proportional to the membrane thickness. However, when a membrane is thin, a large amount of heat will be transferred by conduction through the membrane, leading to low heat efficiency of the DCMD process. Therefore, a compromise should be made between the mass and the heat transfer by properly adjusting the membrane thickness [1,6]. To overcome this disadvantage, a dual-layer hollow fiber consisting of a fully finger-like macrovoid inner-layer and a sponge-like outer-layer was proposed [5]. 

During water desalination the scaling phenomenon should be expected, which increases a risk of membrane pore wetting. Therefore, the thicker membranes, besides their lower process efficiency, may be more accurate for industrial applications. In this work the influence of process parameters and membrane morphology on the effectiveness of desalination by the MD process was studied. Polypropylene capillary membranes with different diameters and pore sizes were used. The changes in MD process efficiency during long-term water desalination were analyzed. 

### 1.1. Mass and Heat Transfer

**Figure 1:**
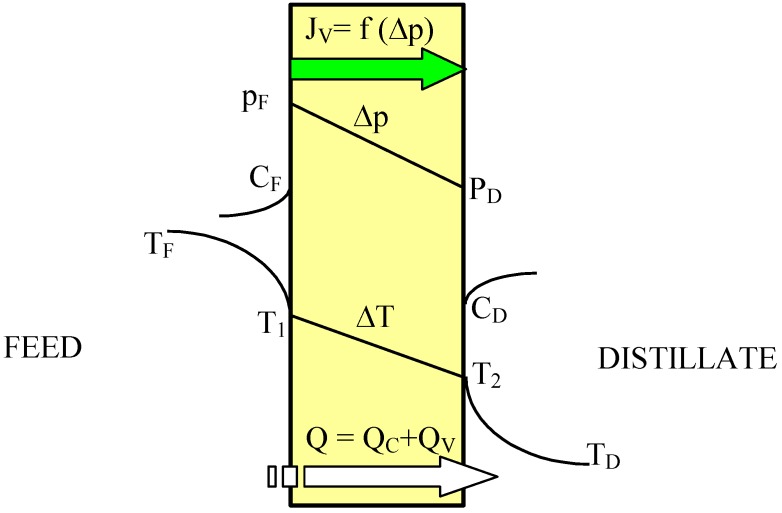
The heat and mass transfer in the DCMD variant of MD process.

The driving force of the mass transfer in the MD process is a vapor pressure difference on both sides of the membrane, which depends on the temperature and solution compositions in the layers adjacent to the membrane (Figure 1). The permeate flux can be described by [2,11]:

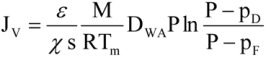
(1)
where p_F_ and p_D_ are the partial pressures of saturated water vapor at temperatures T_1_ and T_2_, and *ε, χ*, s, T_m_ are the porosity, tortousity, thickness, and mean temperature of the membrane, respectively, and M is the molecular weight of water, R is the gas constant, P is the total pressure, and D_WA_ is the effective diffusion coefficient of water vapor through the membrane pores. 

According to Equation 1 the permeate flux increases along with an increase in the membrane porosity and pore diameter, and when the membrane wall thickness is reduced. However, the thin membranes can undergo mechanical damage and become wet more easily [6,9,12]. In MD process mass transfer (J_V_) occurs simultaneously with heat flux (Q) across the membrane, and, as a result, the temperature of the boundary layer on the feed side is lower, whereas on the distillate side it is higher than that of the bulk (Figure 1). This phenomenon is termed temperature polarization [1]. It causes a decrease of the vapor pressure difference across the membrane, which leads to a reduction of the magnitude of mass flux (permeate) flowing through the membrane. Therefore, the permeate flux can be increased several times when the flow rate and temperature of the feed is enhanced in an appropriate way. Moreover, the shape and dimensions of the channels through which liquid flows in the module has an important effect on the permeate flux [1,2,6].

**Figure 2:**
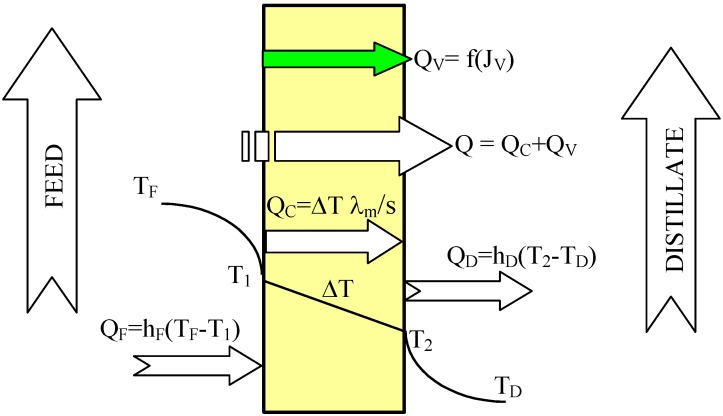
Heat transfer in the DCMD process.

The heat transfer through the membranes leads to cooling of hot feed as well as to heating of the distillate inside the MD module. Therefore, in the DCMD process, it is necessary to supply heat to the hot stream and to remove heat from the distillate stream. The heating and cooling steps represent the energy requirements of the DCMD process. The heat transfer inside the membrane (Q–total heat) takes place by two possible mechanisms: as conduction across the membrane material and its gas-filled pores (Q_C_), and as latent heat associated with vapor flowing through the membrane (Q_V_)-Figure 2. The heat used effectively in the DCMD is the energy consumed as the latent heat for water vapor production, whilst the heat transferred by conduction across the membrane is considered as a heat loss. The heat efficiency (η_T_) in the MD process can be defined by Equation 2 [1,2,11]:

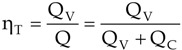
(2)

The amount of heat exchanged in the MD module increases along with an increase of feed temperature [1]. However, under these conditions the permeate flux also increases, which causes the limitation of heat losses (heat conducted through the membrane material). As a result, an increase in the module yield influences the enhancement of heat efficiency of the MD process, and the amount of heat conducted across the membrane (Q_C_) decreases [1,2,9,11]. The heat conducted through the membrane material can be calculated from the following Equation:

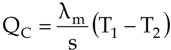
(3)
where s is the membrane thickness.

The thermal conductivity coefficient (λ_m_) for membranes utilized in the MD changes is in a small range: 0.04–0.06 W/mK, and can be determined on the basis of the membrane material data [1]:

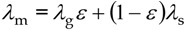
(4)
where λ_g_ is the thermal conductivity of gas phase inside the membrane pores, and λ_S_ is the thermal conductivity of polymeric material. The reported thermal conductivities of PVDF material are within the range 0.17–0.21 W/mK, while those reported for PTFE are 0.25–0.29 W/mK and 0.11–0.2 W/mK for PP [1].

The temperatures in the layer adjacent to the membrane (T_1_, T_2_), depend upon the values of the convective heat transfer coefficients (h_i_) in the MD module. The values of coefficient h_i_ can vary in a wide range, depending on both the design and working conditions of the MD module. Thus, the accurate determination of its value has an essential meaning. If the heat transfer through the feed as well as the distillate boundary layers is very high, the interfacial temperatures (T_1_ and T_2_) approach those of the bulk phases (T_F_ and T_D_, respectively). The coefficients h_D_ and h_F_ can be calculated from the Nusselt correlations [1]. For a module with the membranes arranged in the form of braided capillaries the following form of the Nusselt number can be used [11]:

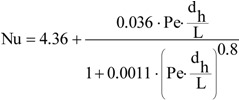
(5)
where L is the module length, d_h _isthehydraulic diameter, the Peclet number is given by the equation Pe = Pr Re, the Prandtl number is Pr = c_P _μ/λ, the Reynolds number is Re = v d_h_ ρ/μ and μ, ρ, c_P_ are the viscosity, density and the specific heat of solution, respectively.

**Figure 3:**
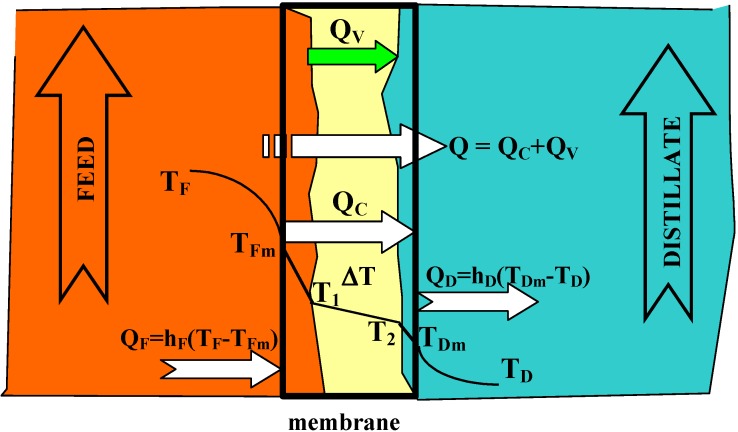
The heat transfer in the MD module with partially wetted membranes.

The heat and mass transfer conditions described above are appropriate when we used a new non-wetted membrane. However, during long-term MD module exploitation the used membranes are partially wetted [2,6], which can significantly influence MD process efficiency. The water-filled layers of surface pores created the additional resistance for heat transfer, and as a consequence, the temperature of the evaporation surface decreases (Figure 3). Due to the wetting phenomenon, the thermal conductivity coefficient of the wetted membrane will be changed:

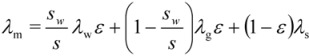
(6)
where λ_w_ is the thermal conductivity of water inside the membrane pores, and s_W_ is the thickness of membrane layer filled with water. 

If water filled the pores e.g., Accurel PP S6/2 membrane at a depth of 100 μm, according to Equation 6, we have λ_m_ = 0.173 W/mK (λ_w_ = 0.675 W/mK). For comparison, the λ_m_ coefficient calculated for dry membranes is equal to 0.059 W/mK. This result indicated that a significant influence of the wetting phenomenon on the MD process efficiency might be expected.

## 2. Experimental Section

**Figure 4:**
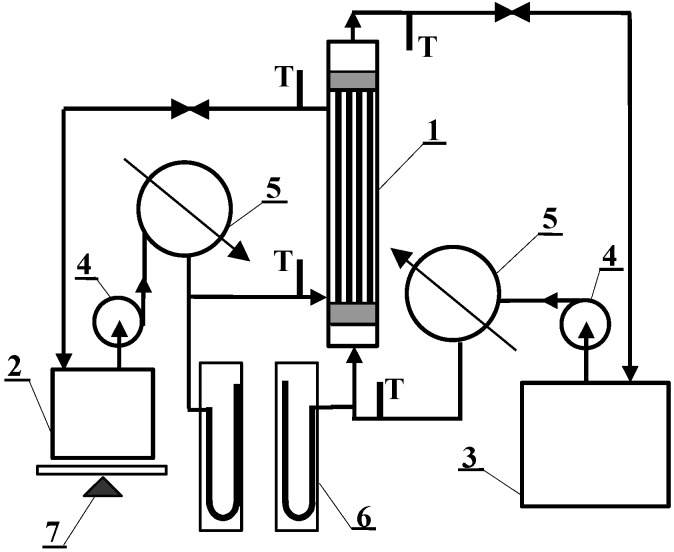
MD experimental set-up: 1: MD module, 2: distillate tank, 3: feed tank, 4: pump, 5: heat exchanger, 6: manometer, 7: balance, T: thermometer.

MD investigations were carried out using the experimental set-up shown in Figure 4. The installation consists of two thermostatic cycles (feed and distillate) connected to a membrane module (inner diameter D = 1.2 cm). The module was assembled in a vertical position in the MD installation. The feed and distillate streams flowed concurrently from the bottom to the upper part of the MD module. The design of the MD module enables the replacement of cartridges with capillary membranes. Polypropylene Accurel PP capillary membranes with different parameters, listed in Table 1, were used for MD investigations. In the case of Accurel PP V8/2 HF, a larger housing of module were used: (a) L = 71 cm and D = 1.2 cm; (b) L = 58 cm and D = 2.2 cm. During the experiments, the feed was supplied inside the capillaries (0.0075–0.014 dm^3^/s), whereas the distillate flowed on the shell-side of the MD module (0.008–0.014 dm^3^/s). The study was also carried out with an Accurel PP V8/2 HF membrane, in which a reversal flow was applied. The inlet temperature of distillate (293 °K) was constant during all experiments, while the feed temperatures were varied in the range of 333–358 °K. The stream temperatures were measured using the thermometers with a ±0.2 K accuracy.

**Table 1:** Parametersof investigated capillary membranes and modules.

Membrane	*d*/mm	*s*/mm	*ε*/%	*d*_P_/µm	*n*	*L/m*
Accurel PP S6/2	1.8	0.40	73	0.2	10	0.25
Accurel PP S6/4	1.6	0.44	73	0.4	10	0.25
Accurel PP Q3/2	0.6	0.20	73	0.2	77	0.25
a) Accurel PP V8/2 HF	6.0	1.3	73	0.2	1	0.71
b) Accurel PP V8/2 HF	6.0	1.3	73	0.2	3	0.5

*d*: Capillary inside diameter; *s*: wall thickness; *ε*: porosity; *d*_P_: nominal pore diameter; *n*: number of membranes inside the shell, *L*: working length of membranes.

The temperature measurement for one-side only flow was carried out for evaluation of heat loss to the environment. The difference between inlet and outlet temperature was below 0.5 K (feed) and close to zero for distillate. Therefore, the heat loss to the environment was almost eliminated when the distillate flowed on the shell-side of the MD module.

The heat efficiency (η_T_) was calculate using the Equation:

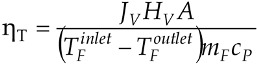
(7)
where T_F_^inlet^ and T_F_^outlet^ are the feed temperature at the MD module inlet and outlet, H_V_ is latent heat of evaporation, m_F_ is feed mass flow, and A is module area, respectively.

The membrane morphology was studied using a Jeol JSM 6100 scanning electron microscope (SEM). The membrane surface (both internal and external) and the membrane cross-sections were subjected to observations. After the completion of MD investigations, the membranes were washed with distilled water followed by natural drying at room temperature. The samples for cross-section observations were prepared by fracturing the capillary membranes in liquid nitrogen. All the samples were sputter coated with gold and palladium.

## 3. Results and Discussion

**Figure 5:**
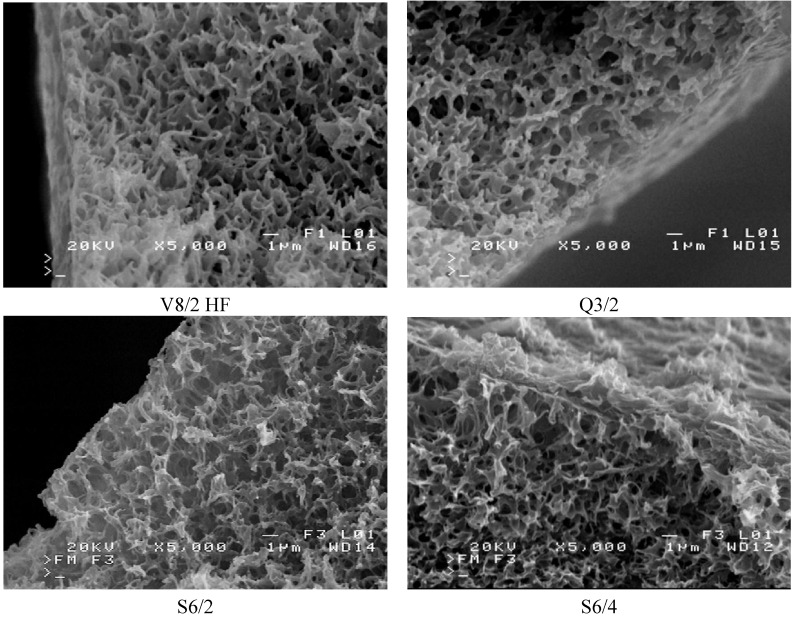
SEM images of cross section of Accurel PP membranes.

**Figure 6:**
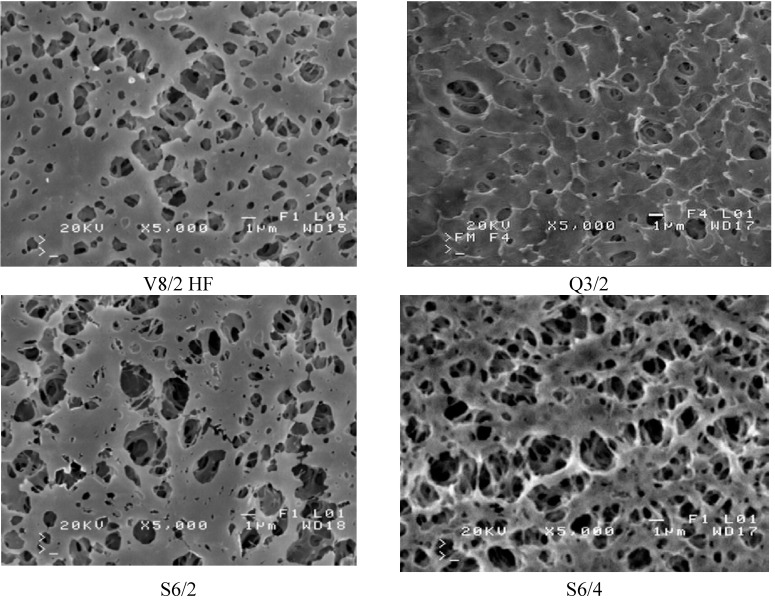
SEM images of Accurel PP membrane surfaces (bore side).

The SEM observation indicated that the studied Accurel PP membranes are characterized by a foam structure and they are symmetrical. Some differences in the pore size occurred only on the capillary external surfaces (Figure 5 and Figure 6). The maximum pores size observed on the internal surface of the capillary membranes did not exceed a few micrometers.

**Figure 7:**
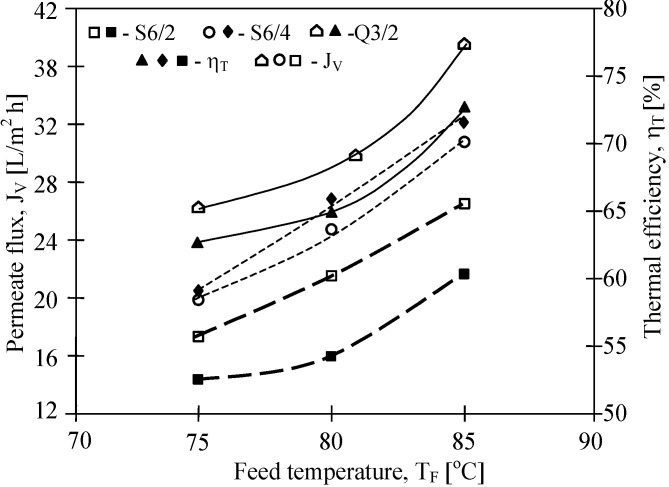
The influence of feed temperature and kind of used membranes on the permeate flux and thermal efficiency. Accurel PP capillary polypropylene membranes: S6/2, S6/4 and Q3/2. Feed–distilled water. T_D_=20 °C, m_F_=m_D_=0.014 dm^3^/s.

**Figure 8:**
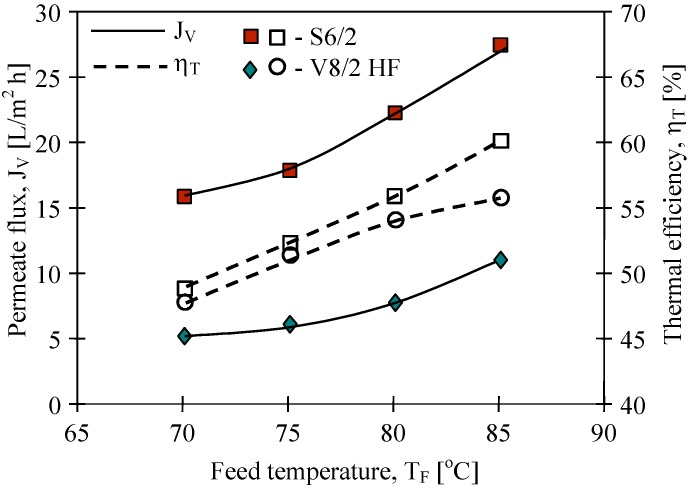
The influence of feed temperature and kind of used membranes on the permeate flux and thermal efficiency. Accurel PP capillary polypropylene membranes: S6/2 and V8/2 HF (0.58 cm-Table 1). Feed–distilled water. T_D_= 20 °C, m_F_=m_D_=0.014 dm^3^/s.

The mass transfer in the MD process is diffusive. Therefore, the permeate flux is strongly affected by membrane wall thickness and pore diameter. The results of the performed study confirmed that the kind of membranes used has a significant influence on MD process efficiency (Figure 7). The highest yield was achieved for the Q3/2 membranes, which had the wall two times thinner (shorter path of diffusion) than the S6/2 and S6/4 membranes. The lowest permeate flux was obtained for the V8/2 HF membranes (Figure 8), having the wall thickness 3–6 times larger than other ones (Table 1). Considering the capillary membranes with a similar wall thickness (S6/2 and S6/4), a higher flux was obtained for S6/4, having a two-times larger pore size than the other membranes. The molecular and Knudsen diffusion influence the mass transfer in MD process, therefore, the permeate flux increases with an increase in the pore diameter.

The driving force for the mass transfer increases with increasing temperature of the feed stream, therefore, the feed temperature has a significant effect on the level of the permeate flux in the MD process. At a constant distillate temperature (20 °C), for all kinds of membranes used, the elevation of T_F_ from 75 °C to 85 °C caused an increase in the permeate flux by about 60%–80% (Figure 7 and Figure 8). Such a high increase of the permeate flux can be explained by an exponential dependence of the vapor pressure on temperature. The results presented confirmed that 50%–80% energy is consumed as latent heat for permeate production, and a higher thermal efficiency in the MD process could be achieved when the feed temperature is close to the boiling point. A decrease of the membrane wall thickness increases the amount of heat conducted by membrane material. However, under these conditions the permeate flux also increases, which causes a limitation of heat losses. As a result, the thermal efficiency is higher when membranes with a thinner wall are used. This effect is reduced when the MD process with membranes having thicker wall is carried out (Figure 8).

**Figure 9:**
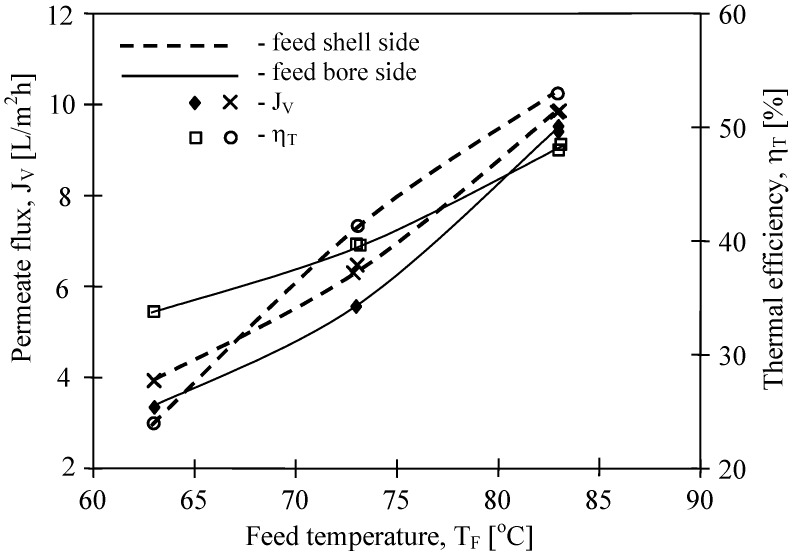
The influence of feed temperature and flow side on the permeate flux and thermal efficiency. Membrane Accurel PP V8/2 HF (0.71 cm—Table 1). T_D_= 20 °C, m_F_=m_D_=0.014 dm^3^/s.

According to Equation 3, the use of the membranes with larger wall thicknesses can decrease the amount of heat conducted from feed to distillate. The Accurel PP V8/2 HF membranes have a wall thickness about 1.3 mm, thus almost three times higher than the wall of S6/2 and S6/4 membranes (Table 1). Although the thick wall restricts the heat losses, it also significantly increases the mass transfer resistance (Equation 1). As a result, the permeate flux was significantly smaller than that obtained for the remaining membranes use in this study (Figure 8), and simultaneously, the thermal efficiency also was lower (Figure 9). A slightly higher efficiency was obtained when feed flowed on the shell side of the MD module with V8/2 HF membranes.

The results presented in Figure 9 suggested that the flow rate (shell and bore side) considerably affects the efficiency of the MD process. In general, the efficiency of the MD module increases with an increase in the flow rate. The effect is particularly significant for the feed flow rate, whereas for the distillate flow rate this effect is slightly smaller [4]. The temperatures in the layers adjacent to the membrane (Figure 2—T_1_, T_2_) are dependent on the values of the heat transfer coefficients in the MD module [11]. The values of these coefficients increase with increased flow rates (Equation 5), thus, the temperature polarization can be considerably reduced by the application of high flow rates [1,11]. This causes an increase of vapor pressure difference (p_F_–p_D_), which, in accordance with Equation 1, causes an increase in the permeate flux (J_V_). Moreover, the application of higher flow rates decreases the temperature difference of streams at the inlet and outlet of the module, resulting in the increase of the temperature difference across the membrane. 

**Figure 10:**
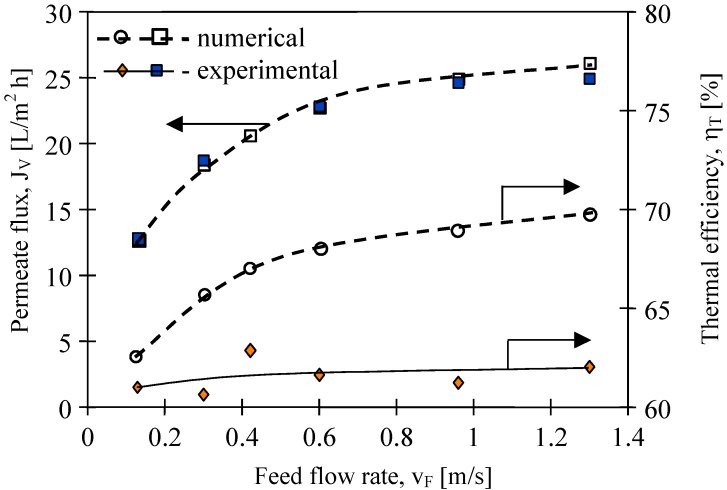
The influence of feed flow rate on the permeate flux and thermal efficiency. Accurel PP S6/2 membranes. Feed—distilled water. T_F_= 80 °C, T_D_= 20 °C.

The results presented in Figure 10 confirmed that the feed velocities had an important role in improving the mass transfer. The application of feed velocity below 0.3 m/s would cause a decrease in the thermal efficiency. Along with the increase, the flow velocity and also the h_F_ and h_D_ values increase, thus the boundary layer temperatures (T_1_ and T_2_) approach the temperatures of the bulk solutions (T_F_, T_D_) [11]. The decline of the temperature polarization besides increasing the permeate flux caused the increase in the driving force for heat transport (Figure 2). 

Moreover, the improvement of module efficiency obtained by the changes of the flow rate is limited, since for each MD module there exists an optimum stream flow rate. The experimental results demonstrate that the efficiency of the MD module used in the studies (Figure 10) approaches a plateau for feed flow rates above 0.5 m/s. Based on the Antoine equation, the permeate flux increases exponentially with temperature, while on the other hand, the conductive heat is affected linearly (Kelvin’s law). Thus, theoretically, the thermal efficiency must be higher for a higher feed velocity. The Equations 1–5 and MD model presented in work [11] were used for numerical simulation of the MD process. The best agreement between numerical and experimental permeate fluxes were obtained (Figure 10). Moreover, the calculation of thermal efficiency based on the numerical results confirmed the theoretical prediction that this parameter increases with flow velocity. However, the values of η_T_ obtained for experimental results are almost constant (about 62%). It is probably due to the fact that the thermal conductivity coefficient (λ_m_) of membranes increases with increases in permeate flux. In this case, an increase of water vapor concentration occurs inside the pores, which facilitated the heat conduction through the membrane wall (Equations 3 and 4).

**Figure 11:**
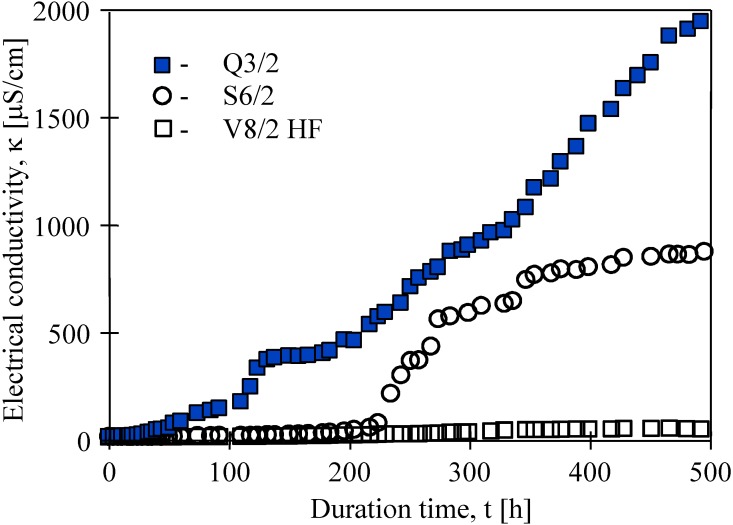
Changes of electrical conductivity of distillate obtained during water desalination by the MD process for different Accurel PP membranes. Feed–salt solution (170 g NaCl/dm^3^). T_F_= 80 °C, T_D_= 20 °C.

Besides thermal efficiency, the scaling and membrane wetting phenomena are very important for the long-term MD process. The studies conducted revealed that permeate flux gradually decreased as a function of operating time of the membrane modules. During almost 500 h separation of NaCl solutions, the permeate fluxes decreased 37%, 25% and 8% for Q3, S6/2 and V8/2 HF membranes, respectively. Simultaneously, the distillate electrical conductivity increased, which was possible owing to a partial wetting of the capillary membranes (Figure 11). However, for all cases studied, over 99% salt rejection was obtained. In the case of S6/2 membranes, an increase of electrical conductivity from 5.6 μS/cm to 700 μS/cm was observed between 200 h and 300 h of module operation, and a value of about 750 μS/cm was maintained during the next 200 h. This indicates that during the first 300 h, the largest pores and pores close to the membrane surface primarily underwent wettability, whereas, the gaseous phase inside the membrane wall was still retained [6,9]. The best purity of distillate was obtained when V8/2 HF membranes were used. 

The results obtained reveal that Accurel PP V8/2 HF membranes are very resistant to pore wettability. Therefore, for the separation of salt solutions the use of thicker membranes is more advantageous.

**Figure 12:**
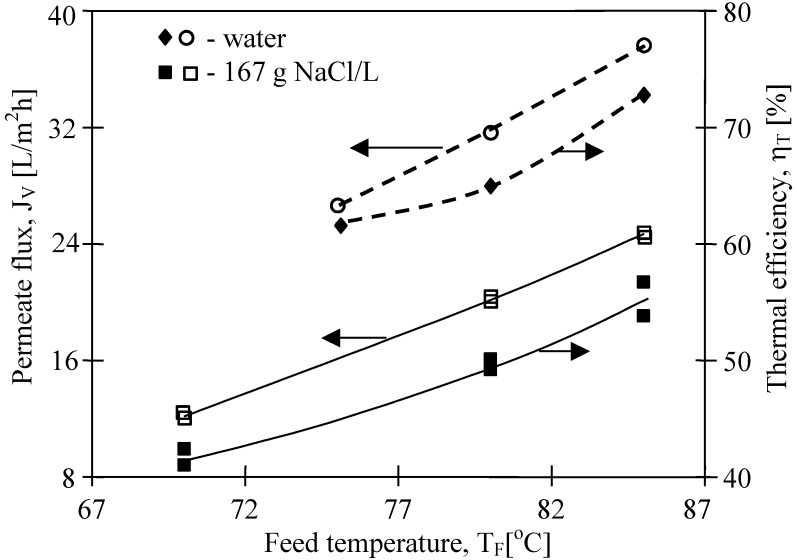
The influence of feed temperature and salt concentration on the permeate flux and thermal efficiency. Accurel PP S6/2. T_D_= 20 °C, m_F_=m_D_=0.014 dm^3^/s.

**Figure 13:**
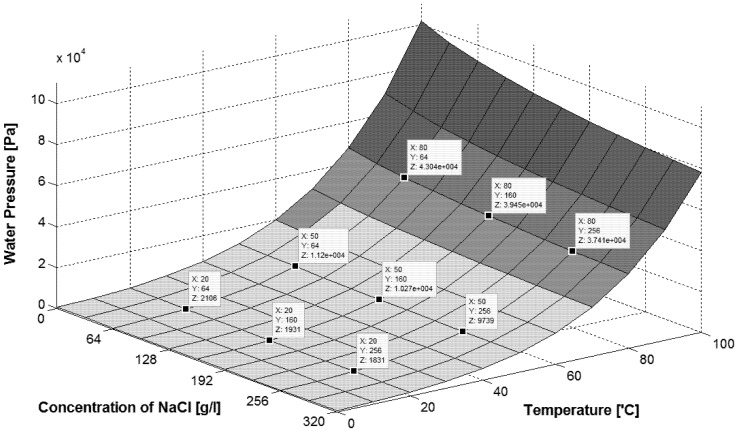
The influence of feed temperature and salt concentration on water vapor pressure. Data for calculation taken from [14].

The effect of salt presence in the feed on permeate flux is shown in Figure 12. The obtained permeate fluxes decrease about 40%, when the salt concentration in the feed increased from 0 g to 167 g NaCl/L. The obtained results confirmed that the increase of solute concentration caused the reduction of the driving force for the mass transfers, resulting in a decline of permeate fluxes [13]. According to the Rault’s law, a growing concentration of solutes in the feed causes a decrease of vapor pressure above the solution and the decrease of the difference (p_F_ − p_D_). This effect is enhanced by concentration polarization, and this coefficient for the MD process conditions was calculated at a level of 1.1 (expressed as c_1_/c_F_) [13]. However, when the salt concentration increases to 320 g NaCl/L, only a 25% degree of permeate flux should be expected (Figure 13). The observed decreases of the permeate flux is probably also influenced by a significant decrease of the convective heat-transfer coefficient with increased solution concentration. 

## 4. Conclusions

Higher thermal efficiency can be obtained when operating conditions that increase permeate flux are used in the MD process. Therefore, the increase of the flow rate and feed temperature caused an increase in the MD process efficiency.

By increasing the thickness of the membrane wall, heat transfer by conduction will be reduced, but simultaneously mass transfer resistance will increase. As a result, the heat efficiency is lower for membranes with higher wall thicknesses.

A decline of permeate flux was found along with an enhancement of salt concentration, and for the concentrated NaCl solution the flux was lower by 40 %, in comparison with that for distilled water. The dissolved salts decrease the water vapor pressure, which reduced the permeate flux and as a result the thermal efficiency is lower during MD process of brines. 
